# A tale of autoimmunity: thymoma, thymectomy, and systemic lupus erythematosus

**DOI:** 10.1007/s10067-020-05061-z

**Published:** 2020-04-10

**Authors:** Arash Mollaeian, Christopher Haas

**Affiliations:** 1 MedStar Health Internal Medicine Residency Program, Baltimore, MD USA; 2 Georgetown University School of Medicine, Washington, DC USA

**Keywords:** Autoimmunity, SLE, Thymectomy, Thymoma, Thymus

## Abstract

The thymus plays an integral role in immune system regulation, modulating the development, diversity, and selection of T lymphocytes, a critical feature for the prevention of T cell-mediated autoimmunity. Thymoma is a rare tumor of the thymus. Autoimmune diseases and paraneoplastic syndromes such as myasthenia gravis, pure red blood cell aplasia, and systemic lupus erythematosus, although relatively uncommon, have been described in association with thymomas. Rare cases of post-thymectomy autoimmune related diseases, including systemic lupus erythematosus and pure red cell aplasia, have been reported in the literature. Here, we present the case of a 65-year-old male who developed systemic lupus erythematosus 2 years after thymectomy in the setting of thymoma-associated pure red cell aplasia.

## Introduction

The thymus is a mediastinal lymphoid organ that plays a significant role in development and maintenance of cell-mediated immunity and central tolerance, controlling the differentiation and maturation of naïve T lymphocytes into helper (CD4) and cytotoxic (CD8) lymphocytes, T lymphocyte migration to peripheral tissues, and secretion of regulatory hormones throughout early development through late adulthood [1–3]. Thymomas are rare epithelial tumors of the thymus, which are associated with autoimmune and paraneoplastic syndromes. The pathogenesis of thymoma-induced autoimmunity is not well understood; however, various theories have been postulated, all of which relate to dysregulation of normal thymus function and physiology. Myasthenia gravis (MG) is the most common autoimmune disorder associated with thymoma, and 10–15% of MG cases have thymoma, while up to 50% of thymoma cases may develop MG. The incidence of SLE associated with thymoma is far less and as low as 1.5% [1]. Thymectomy is an established treatment strategy for thymoma and other thymoma-associated autoimmune diseases such as myasthenia gravis and pure red cell aplasia (PRCA). Interestingly, while thymectomy has been reported to be associated with decreased symptomatology of underlying autoimmune disease [2], there are reports not only of de novo autoimmune disease, but worsening of underlying disease post-thymectomy, specifically systemic lupus erythematosus (SLE) [1].

SLE is a systemic autoimmune disease with variable clinical features, disease course, and prognosis. The diagnosis and management of SLE remains a clinical challenge for physicians. The diagnostic criteria for SLE have evolved, with the 1997 revised American College of Rheumatology (ACR) criteria and 2012 Systemic Lupus International Collaborating Clinics (SLICC) criteria commonly accepted, relying on a combination of clinical and laboratory findings. Recently, SLE diagnostic and classification criteria were updated by the American College of Rheumatology (ACR) and the European League Against Rheumatism (EULAR) [13]. Here, we present the case of a 65-year-old male with thymoma and associated PRCA who developed features of SLE 2 years post-thymectomy.

## Case presentation

**Fig. 1:**
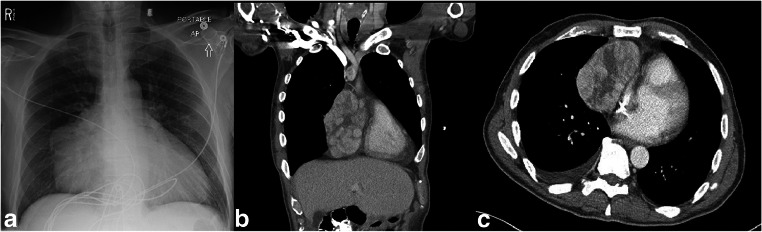
**a** Chest X-ray before thymectomy and thymomectomy. **b**, **c** Chest CT scan: right-sided, anterior mediastinal well-circumscribed mass without evidence of gross infiltration or necrotic, cystic, or calcified components

A 65-year-old African-American never-smoker vegan male with a past medical history of recurrent, transfusion-dependent anemia of unknown etiology of four years duration presented to an outside hospital with the chief complaint of abdominal pain. The review of the workup for his transfusion-dependent anemia revealed a severe normocytic, non-hemolytic anemia with iron studies indicative of anemia of chronic disease. Folate, cyanocobalamin, and thyroid stimulating hormone levels were normal (Table 1). Marrow biopsy failed to demonstrate any evidence of aplastic anemia or myelofibrosis, and the patient was given a presumptive diagnosis of PRCA, although no specific myeloid to erythroid ratio was available. On presentation to the outside facility, he was incidentally found to have a large mediastinal mass on chest X-ray (Fig. 1a), which was confirmed by computed tomography (CT) scan. CT imaging revealed a 11.7 × 9.8 × 7.6 cm right-sided, anterior mediastinal well-circumscribed mass without evidence of gross infiltration or necrotic, cystic, or calcified components, though it did deform the pericardium from extrinsic compression (Fig. 1b, c). Pathology from a CT-guided biopsy revealed thymoma. The patient was evaluated by cardiothoracic surgery who deemed the mass unresectable given its large size and tortuous anatomy, advising neoadjuvant chemotherapy for interim management. Anti-nuclear and acetylcholine receptor antibodies were negative at the time.

**Table 1:** Laboratory data on first assessment after diagnosis of thymoma

Lab	Value	Reference
WBC	7.7 k/μL	4.0–10.8
Neutrophil	62.9%	43.0–75.0
Lymphocyte	27.9%	15.0–45.0
Monocyte	7.8%	3.0–12.0
Eosinophil	0%	0.0–6.0
Basophil	0.8%	0.0–2.0
Absolute neutrophil count	4.9 k/μL	1.7–8.1
Absolute lymphocyte count	2.2 k/μL	0.6–4.9
Absolute monocyte count	0.6 k/μL	0.1–1.3
Absolute eosinophil count	0.0 k/μL	0.0–0.7
Absolute basophil count	0.1 k/μL	0.0–0.2
Immature granulocyte	0.6%	0.1–0.3
Absolute immature granulocyte	0.05 k/μL	0.01–0.03
Hgb	2.5 g/dL	12.5–16.5
Hct	7.5%	37.5–49.5
MCV	83.3 FL	81.0–100.0
MCH	27.8 pg	27.0–31.0
MCHC	33.3 g/dL	31.0–36.0
RBC	0.90 million/μ L	4.20–5.50
RDW	14.2%	11.5–15.5
Platelet	225 k/μL	145–400
MPV	10.6 FL	1.5–10.4
Absolute immature platelet	11.0%	1.1–6.7
Reticulocyte count	0.2%	0.5–2.0
Absolute reticulocyte count	0.001 million/μL	0.020–0.100
Haptoglobin	295 mg/dL	31–200
Folate	4.9 ng/mL	4.1–55.4
Vitamin B_12_	663 pg/mL	211–911
Iron	209 mcg/dL	49–181
TIBC	246 mcg/dL	261–462
Iron saturation	85%	20–55
LDH	190 units/L	87–241
Haptoglobin	295 mg/dL	31–200

*WBC* white blood cells, *Hgb* hemoglobin, *Hct* hematocrit, *MCV* mean corpuscular volume, *MCH* mean corpuscular hemoglobin, *MCHC* mean corpuscular hemoglobin concentration, *RBC* red blood cells, *RDW* red cell distribution width, *MPV* mean platelet volume, *TIBC* total iron binding capacity, *LDH* lactate dehydrogenase

**Fig. 2:**
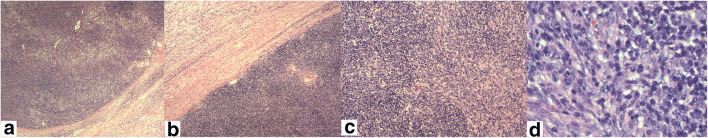
Thymoma biopsy. **a** H&E stained slide at × 20; cellular nodular well-circumscribed tumor with fibrous bands (lower right) and a cellular nodule of tumor. **b** H&E stained slide at × 40; well-circumscribed nodule of tumor with fibrous band (upper left) and tumor nodule (lower right). **c** H&E stained slide at × 100; type AB thymomas have two microscopic components; there is a homogeneous population of neoplastic spindle cells without nuclear atypia (right mid to lower) and scattered foci which are rich in lymphocytes (seen predominantly in left lower and mid portion of image). **d** H&E stained slide at × 400; higher power image with neoplastic spindle cells admixed with lymphocyte rich foci

In consultation with oncology, a chemotherapeutic regimen of cisplatin, cyclophosphamide, and adriamycin commenced. In the months following initiation of chemotherapy, the patient’s red blood cell transfusion requirements decreased and an interval CT scan demonstrated a gradual reduction in the size of his thymoma to 9 × 5.5 × 11.5 cm. At that time, cardiothoracic surgery re-evaluated the patient and subsequently performed a complete thymectomy and thymomectomy. Gross and microscopic histopathology revealed benign thymus tissue with WHO class AB and modified Masaoka stage I thymoma, indicative of a well-circumscribed, grossly and microscopically encapsulated, noninvasive mass with characteristic lymphocyte-rich and lymphocyte-poor areas (Fig. 2a–d).

**Fig. 3:**
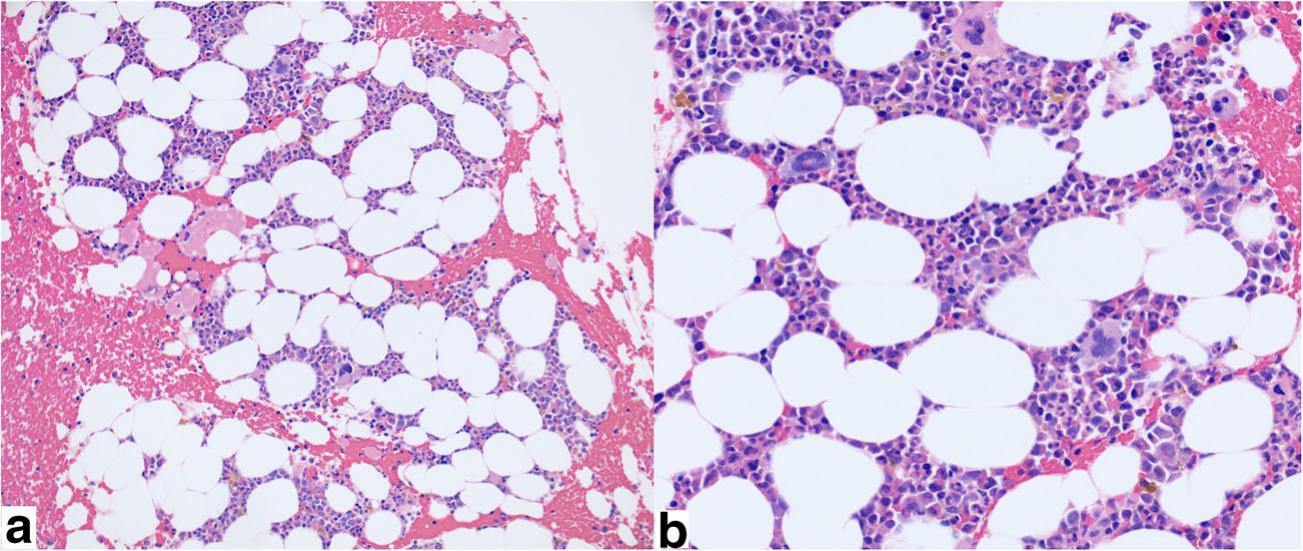
Bone marrow biopsy. **a**, **b** Hypocellular marrow (20–30%) with difficult to identify erythroid precursors and a myeloid-erythroid ratio of greater than 10:1. Vast majority of cells are in the myeloid series with many mature neutrophils. Scattered megakaryocytes are present

Despite initial improvement in his hemoglobin and transfusion requirements (transfusion threshold increased to Hgb of 6) following chemotherapy and thymectomy, the patient nevertheless remained severely anemic with a continued normocytic, non-hemolytic anemia, dependent on weekly red blood cell transfusions. He received a total of 150 red blood cell transfusions during a period of almost 40 months; however, there was no evidence of alloimmunization. Given his persistent anemia, he underwent repeat bone marrow biopsy 6 months post-thymectomy, which subsequently revealed a hypocellular marrow (20–30%) with difficult to identify erythroid precursors and a myeloid-erythroid ratio of greater than 10:1, indicative of erythroid hypoplasia. Megakaryocytes were normal in number and morphology, and occasional paratrabecular aggregates of small, mature lymphocytes were observed (Fig. 3a, b). Flow cytometry was negative for lymphoma, revealing no loss or aberrant expression of T lymphocyte cell antigens, a normal CD4/CD8 ratio, no evidence of monotypic population of B lymphocytes, and no increased blasts. He was referred for evaluation of bone marrow transplant, however declined further workup.

His transfusion dependency was subsequently complicated by iron overload, diagnosed in the setting of iron saturation of 93% and ferritin elevation up to 6000 ng/ml, for which deferasirox was initiated. He eventually acquiesced to a bone marrow transplant workup. Repeat marrow continued to demonstrate a hypocellular marrow with scant erythroid precursors and an elevated myeloid-erythroid ratio with normal myeloid lineage maturation, while flow cytometry continued to demonstrate no abnormalities. Whole-body positron emission tomography scan was negative for FDG-avid masses, suggesting no evidence of residual thymoma tissue or metastatic disease. An anti-nuclear antibody (ANA) was positive with a titer of 1:160 with speckled and homogenous patterns, which prompted further autoimmune workup and referral to rheumatology.

At the time of referral to rheumatology, ANA titer remained elevated (1:320) with a predominantly homogenous pattern. Anti-Smith and double-stranded DNA (ds-DNA) antibodies (Ab) were positive, whereas anti-histone Ab, ribonucleic protein (RNP) Ab, RNA polymerase 3 IgG Ab, Ro/SSA and La/SSB Ab, Scleroderma (SCL) 70 Ab, rheumatoid factor (RF), anti-neutrophil cytoplasmic antibody (ANCA), anticardiolipin Ab, thyroid antibodies, and beta-2 glycoproteins antibodies were negative. C3 and C4 complement levels were within normal limits (Table 1). Urine studies showed increased urine protein-creatinine ratio of 663 mg/g. He fulfilled not only the SLICC criteria, but also the EULAR criteria for diagnosis of systemic lupus erythematous (Table 2). It was recommended that he be started on hydroxychloroquine 400 mg daily; however, the patient declined as he did not agree with the diagnosis of lupus and the potential side effects of the proposed medication, despite ongoing discussion with the primary team as well as sub-specialty services. Hematology recommended immunosuppressive therapy, prednisone, and cyclophosphamide, to which he agreed. A comprehensive infectious workup prior to initiation of immunosuppression was unremarkable, and he was started on prednisone for 1 month to be followed by cyclophosphamide. In the setting of his aforementioned oral ulcers and oral dysphagia, however, he was unable to tolerate cyclophosphamide and further declined to pursue that option again. Other therapeutic options including methotrexate and cyclosporine were declined in the setting of adverse side effect profiles, and the patient decided to remain on intermittent palliative transfusions.

**Table 2:** SLICC and ACR/EULAR lupus classification criteria

SLICC criteria		Fulfilled criteria by patient	
Acute cutaneous lupus			
Chronic cutaneous lupus			
Oral ulcers		Present	
Nonscarring alopecia			
Synovitis			
Serositis			
Renal		Proteinuria (663 mg/g)	
Neurologic			
Hemolytic anemia			
Leukopenia		Present (3.4 k/μL)	
Thrombocytopenia		Present (< 50 k/μL with nadir of 5 k/μL)	
ANA above lab reference range		Present (1:320)	
Anti-Ds-DNA		Present	
Anti-Smith			
APLA			
Low complements			
Direct Coombs in absence of hemolytic anemia			
2019 EULAR/ACR SLE criteria [9]		Fulfilled criteria by patient	
Domain	Item	Score	
Constitutional	Fever	2	
Hematologic	Leukopenia	3	
	Thrombocytopenia	4	4
	Autoimmune hemolysis	4	
Neuropsychiatric	Delirium	2	
	Psychosis	3	
	Seizures	5	
Mucocutaneous	Non-scarring alopecia	2	
	Oral ulcers	2	2
	Subacute cutaneous/discoid lupus	4	
	Acute cutaneous lupus	6	
Serosal	Pleural or pericardial effusion	5	
	Acute pericarditis	6	
Musculoskeletal	Joint involvement	6	
Renal	Proteinuria > 0.5 g/24 h	4	4
	Renal biopsy class II or V lupus nephritis	8	
	Renal biopsy class III or IV lupus nephritis	10	
Antiphospholipid Ab	ACA or anti-β2GP1 Ab or lupus anticoagulant	2	
Complement proteins	Low C3 or C4	3	
	Low C3 and C4	4	
SLE-specific Ab	Anti-ds-DNA Ab or anti-Smith Ab	6	6
Total score			16

As his disease course continued, he gradually developed worsening thrombocytopenia (nadir of 5) and leukopenia, which was complicated by multiple hospitalizations in the setting of opportunistic infections with multi-drug resistant organisms such as extended spectrum beta-lactamase (ESBL) pneumonias and bacteremia. Multiple screening for other underlying causes of immunosuppression such as human immunodeficiency virus (HIV) infection were negative. Bone marrow biopsy and flow cytometry persistently failed to reveal any abnormality in platelet or lymphocyte precursors, with persistent erythroid hypoplasia. Unfortunately, the patient continued to decline immunosuppressive therapy, leading to an insidious decline in his clinical status, ultimately resulting in his death secondary to multi-drug resistant infections with ESBL organisms in the setting of pancytopenia.

## Discussion

The thymus is critical in recognizing and eliminating T lymphocytes that possess the ability to attack self-antigens, in turn preventing autoimmunity by developing self-tolerance. This tolerance to self-antigens occurs in both the thymus and in the periphery, or in the fetal liver and bone marrow, for T and B lymphocytes, respectively [10]. Central tolerance develops via clonal detection of naïve self-reactive T lymphocytes, also known as positive selection, which takes place during the maturation of CD4/CD8 dual-positive T lymphocytes. During this process, T lymphocyte precursors with self-reactivity are positively selected in the thymic cortical and medullary epithelium via interaction with MHC complexes and undergo clonal deletion via apoptosis, whereas those with low avidity for self migrate to peripheral tissues, resulting in survival of only those T lymphocytes with low or absent affinity for self-antigens [3–6, 10]. For those self-reactive lymphocytes that escape clonal deletion in the thymus initially, and mature into CD4 or CD8 positive T lymphocytes with minimal or moderate self-reactivity, the thymus and peripheral tissues have a secondary fail-safe, known as negative selection, during which auto-reactive peripheral T lymphocytes undergo apoptosis in both tissues [4]. While it was initially believed that thymus’ physiologic role in T lymphocyte development disappeared with its involution in adulthood, it is now widely accepted that even though the thymus is largely replaced by fatty tissue and undergoes significant lymphocyte depletion, it continues to play a significant role in maturation and differentiation of T lymphocytes as well as central and peripheral tolerance [2, 4].

Thymic epithelial tumors are comprised of two different tumor types including thymomas and thymic carcinomas [9, 18]. While thymomas are rare, they remain the most common mediastinal mass in adults, comprising up to 25% of mediastinal masses [2]. In cases of thymic hyperplasia or thymoma-associated autoimmune disease (e.g., myasthenia gravis, pure red cell aplasia), thymectomy has been considered standard of care [1–6]. Thymomas are classified and staged based on the World Health Organization (WHO) class system and Masaoka stage, respectively [1, 2]. The WHO classification system focuses on the location and primary cellular components of the thymic tumor and are classified into types A, B, and AB with thymic tissue comprised of the cortical, medullar, or mixed components, respectively [7] (Table 3). In contrast, the Masaoka stage is based on the tumor’s surgical (macroscopic) and pathological (microscopic) extension into surrounding tissues [8] (Table 3).

**Table 3:** WHO classification and Masaoka staging of thymoma [7, 8]

WHO class	Description
A	A tumor composed of a population of neoplastic thymic epithelial cells having spindle/oval shape, lacking nuclear atypia, and accompanied by few or no nonneoplastic lymphocytes
AB	A tumor in which foci having the features of type A thymoma are admixed with foci rich in lymphocytes
B1	A tumor that resembles the normal functional thymus in that it combines large expanses having an appearance practically indistinguishable from normal thymic cortex with areas resembling thymic medulla
B2	A tumor that resembles the normal functional thymus in that it combines large expanses having an appearance practically indistinguishable from normal thymic cortex with areas resembling thymic medulla
B3	A type of thymoma predominantly composed of epithelial cells having a round or polygonal shape and exhibiting no or mild atypia. They are admixed with a minor component of lymphocytes, resulting in a sheetlike growth of the neoplastic epithelial cells
C	A thymic tumor exhibiting clear-cut cytologic atypia and a set of cytoarchitectural features no longer specific to the thymus, but rather analogous to those seen in carcinomas of other organs. Type C thymomas lack immature lymphocytes; whatever lymphocytes may be present are mature and usually admixed with plasma cells.
Masaoka stages	Description
I	Macroscopically completely encapsulated and microscopically no capsular invasion
II	Macroscopic invasion into surrounding fatty tissue or mediastinal pleura or microscopic invasion into capsule.
III	Macroscopic invasion into neighboring organs, i.e., pericardium, great vessels, or lung
IVa	Pleural or pericardial dissemination
IVb	Lymphogenous or hematogenous metastasis

Compared to thymic carcinomas, thymomas are more frequently associated with autoimmune disease and paraneoplastic syndromes including but not limited to myasthenia gravis, PRCA, SLE, bullous pemphigoid, polymyositis, and syndrome of inappropriate anti-diuretic hormone [1, 2, 13, 18]. While it is well-recognized that these diseases may occur in the absence of a thymoma, the presence of a thymoma has been associated with a worse prognosis, which in some part, appears to be dependent on thymoma WHO classification and Masaoka staging [1–3, 9, 10]. In a large retrospective analysis, Padda et al. noted that among different WHO thymoma classes, class B2 is most commonly associated with autoimmune and paraneoplastic disorders, whereas class AB thymomas are least commonly associated [9]. Furthermore, they noted that the presence of an autoimmune or neoplastic syndrome was not an independent prognostic factor for recurrence-free and overall survival in thymic epithelial tumors. Notably, irrespective of WHO classification, independent prognostic factors that portended a worse overall survival include older age, non-thymoma thymic malignancies, advanced Masaoka stage (III–IVB), larger size, and incomplete resection of the tumor [9].

The prevalence of autoimmune disorders/paraneoplastic syndromes in individuals with thymoma or thymic hyperplasia has been reported to be as high as 30%, whereas the prevalence in the general population is in the range of 7–9% [1–6, 12]. The prevalence and incidence of autoimmune disease/paraneoplastic syndromes post-thymectomy is much less characterized; however, reports have shown that it is as high as 8% [1]. The prevalence of SLE in patients with thymoma is reported to be up to 2%; however, this is variable in post-thymectomy cases [1, 11]. Multiple theories on the pathogenesis and development of autoimmune disease/paraneoplastic syndromes in the context of thymoma have been postulated, primarily focusing on an underlying dysregulation of normal thymic function, in turn affecting positive and negative selection processes and defects in central tolerance [1–3].

With respect to T lymphocyte maturation and the development of autoimmunity, a variety of theories have been posited. Typically, progenitor CD4+/CD8+ T lymphocytes formed in the bone marrow migrate to the thymus for maturation. Here, immature progenitor CD4+/CD8+ T lymphocytes undergo receptor gene rearrangement and expression of specific cell surface markers that will allow for the development of a broad antigenic repertoire. These naïve T lymphocytes enter the thymic cortex and migrate to medullary regions for further priming where the process of positive selection, and subsequently negative selection, occurs [2]. The Escape theory, or the immature T cell theory, postulates that autoimmune disease stems from immature T lymphocytes that emigrate from thymic tissue without undergoing the necessary process of positive selection and maturation, thus leading to a population of T lymphocytes lacking appropriate self-tolerance [1, 2]. Alternatively, neoplastic theory, or genetic theory, postulates that cortical T lymphocytes with high proliferation rates (particularly those within thymomas or within thymic hyperplasia) not only have high rates of proliferation, but also reduced expression of HLA-DR, a necessary MHC class II cell surface moiety required for self-selection [1, 2]. Other theories such as the combined cellular and humeral deregulation theory and autoimmune regulator (AIRE) gene mutation theory have been described, potentially leading to impaired negative selection. The combined cellular and humeral autoimmunity theory entertains the thought that along with autoreactive T lymphocytes’ escape into the periphery, a linking step, leading to activation of humeral immunity through the interaction of CD4 T lymphocytes and B lymphocytes, is required. This theory is supported by the presence of autoantibodies in thymomas associated with myasthenia gravis and other autoimmune diseases [2]. AIRE, an autoimmune regulator gene, is responsible for the expression of self-antigens in the thymus and is normally highly expressed in medullary thymic epithelial cells, mediating the expression of tissue-specific antigens, which in turn is associated with positive and negative selection [5]. Interestingly, up to 95% of thymomas lack AIRE expression, thus leading to impaired positive and negative selection and the hypothesized development of autoimmunity and paraneoplastic syndromes [1, 2, 5].

These theories, however, are more applicable to patients that develop autoimmune disease/paraneoplastic syndromes in the context of thymoma rather than those post-thymectomy. We theorize that while de novo T lymphocytes in the post-thymectomy patient are still produced from the bone marrow, they fail to undergo the necessary thymic priming to develop into functional T lymphocyte subtypes and subsequently undergo cell death. In contrast, pre-existing populations of T lymphocytes that have escaped positive and negative selection, likely persist, with minimal self-reactivity. Thymectomy allows for removal of an additional fail-safe, negative selection, leading to the development of autoimmunity. Such a hypothesis is seemingly supported by additional studies. For example, Gerli et al. evaluated the development of select autoimmune diseases in individuals whom underwent thymectomy in the context of thymoma-associated myasthenia gravis. In these patients, T lymphocyte lymphopenia was associated with hypergammaglobulinemia and B lymphocyte hyper-reactivity, and higher titers of auto-antibodies including ds-DNA Ab were observed, demonstrating a direct link between cellular and humoral immunity. They concluded that thymectomy has a potential association with autoimmune disease due to the development of unregulated autoreactive and regulatory T lymphocytes, with subsequent dysregulated B lymphocyte populations with self-reactivity [3]. Furthermore, studies of T lymphocytes in patients with thymoma have suggested that thymomas potentially produce antigen-specific T lymphocytes that migrate from the thymus into peripheral tissues that persist even after thymectomy and potentially stimulate B lymphocytes to produce different antibodies leading to variety of paraneoplastic and autoimmune diseases [14].

The exact mechanism and pathogenesis of autoimmunity and paraneoplastic disease after thymectomy, its prevalence, and predisposition in certain patients remains to be elucidated. Bernard et al. concluded that no clinical nor pathological features predicted the development of autoimmune disorders post-thymectomy and that preexisting autoimmunity was not identified as a risk factor for autoimmunity post-thymectomy [1]. Several cases of thymoma-associated PRCA and/or SLE, as well as their incidence post-thymectomy, have been reported in the medical literature [15–24]. In our case, the patient had a WHO class AB and Masaoka stage I thymoma associated with PRCA and late post-thymectomy development of SLE. PRCA has been reported in about 10% of thymoma cases, and thymectomy has been considered as the treatment of choice in these cases; however, only 25–30% of cases respond to this treatment [1–3, 18]. In this case, our patient’s PRCA initially improved with chemotherapy, however subsequently failed to respond to thymectomy, and ultimately was complicated by post-thymectomy SLE, which, in turn, was further complicated by progressive pancytopenia. He was offered immunosuppressive therapy, which he was unable to tolerate, and eventually declined any further treatment.

In the most recent and comprehensive review of cases of thymoma-associated SLE, Jamilloux et al. [18] reviewed 51 cases and noted that the overwhelming majority of cases (84%) were observed in women with a staggering 42% of cases presenting after thymectomy. The most common presentation reported was articular involvement followed by skin manifestations and serositis, manifestations which have been highlighted by multiple other case reports [14–24]. Our patient developed SLE 2 years after thymectomy. In contrast to the case reports described above, he failed to demonstrate “classic” post-thymectomy SLE manifestation, with a notable lack of articular or skin involvement, with oral ulceration as his main physical manifestation. He nevertheless fulfilled both the SLICC and the EULAR diagnostic criteria for SLE-positive ANA, ds-DNA, anti-Smith antibodies, thrombocytopenia, leukopenia, proteinuria, and oral ulceration [13]. We believed he would benefit from treatment with hydroxychloroquine; however, as mentioned, he declined recommendations for immunosuppressive therapy.

## Conclusion

This case emphasizes an association of thymoma, thymectomy, and multiple autoimmune disorders, particularly PRCA and SLE, and that these disorders can precede or pursue a diagnosis of thymoma. It not only highlights that the development of autoimmune disease and paraneoplastic syndromes post-thymectomy are distinct possibilities, but also underscores the severity of such presentations and the unpredictable nature of the disease course. Larger dedicated and sophisticated studies are required to better understand the underlying mechanism of this association, potential risk factors for development of each autoimmune disorders, and investigation of whether it is reasonable to screen for such disorders in cases of thymoma or vice versa. We suggest the possibility of development of a unique globally accepted diagnostic approach for thymoma and thymectomy-associated autoimmune and paraneoplastic syndromes, to help develop potentially beneficial management strategies in such cases.
